# Mechanisms of Zhenwu decoction for the treatment of renal fibrosis at various stages: What is the role of *Corynebacterium*?

**DOI:** 10.3389/fmicb.2022.913465

**Published:** 2022-09-06

**Authors:** Lijing Du, Yiping Zhang, Shuai Ji, Leqi Wang, Xiaoshan Zhao, Shikai Yan, Xue Xiao, Shasha Li

**Affiliations:** ^1^The Second Clinical College of Guangzhou University of Chinese Medicine, Guangzhou, China; ^2^School of Pharmacy, Shanghai Jiao Tong University, Shanghai, China; ^3^School of Traditional Chinese Medicine, Southern Medical University, Guangzhou, China; ^4^Institute of Traditional Chinese Medicine, Guangdong Pharmaceutical University, Guangzhou, China; ^5^State Key Laboratory of Dampness Syndrome of Chinese Medicine, Guangzhou, China

**Keywords:** Zhenwu decoction, renal fibrosis, intestinal microbiota, *Corynebacterium*, inflammatory

## Abstract

Many studies demonstrated that Zhenwu decoction (ZWD) is effective in the treatment of kidney fibrosis, whereas the mechanism remains unclear. In this work, a microbiomics-based strategy was used to investigate the mechanism of protective effects of ZWD on kidney fibrosis. Unilateral ureteral obstruction was used to replicate a rat model of renal fibrosis, and rats were divided into prophylactic, early, and progression stages according to the timing of administration. Feces was collected to perform microbiota evaluation by high-throughput 16S DNA sequencing. The results indicated that *Corynebacterium*, *Alistipes*, *Dorea*, and *Lactonifactor* were highlighted as key targeted flora of ZWD in the treatment of renal fibrosis, and their biological functions were related to inflammation, immunity, and renal excretion. Especially, *Corynebacterium* presented a significant positive correlation with the concentration of Cys-C, Scr, and BUN. The studies on the changes in inflammatory cytokines (INF-γ, IL-1β, IL-4, and TNF-α) and immunoglobulin (IgA, IgM, and IgG) confirmed the beneficial effects of ZWD on kidney fibrosis. Therefore, this study confirmed the protective effect of ZWD against renal fibrosis at various disease stages, and its mechanism was associated with re-establishing dysbiosis of the intestinal microbiota, reducing inflammation, as well as regulating immune functions. In particular, *Corynebacterium* may be a key flora in the treatment of renal fibrosis.

## Introduction

Chronic kidney disease (CKD) is an increasing global public health problem that affects over 10% of the population worldwide (Levin et al., 2017; Lv and Zhang, 2019). In the process of chronic injury, the continuous overdeposition of the fibrous matrix destroys the structure of the kidney, decelerates local blood supply, and finally leads to kidney injury (Chawla et al., 2014). Renal fibrosis is the final endpoint of the transition from persistent renal injury to CKD (Venkatachalam et al., 2015). Currently, there are no clinically effective therapies to prevent or reverse the progression of renal fibrosis. The most common clinical therapies recommend renin inhibitors, angiotensin-converting enzyme inhibition, and angiotensin II receptor type 1 blockers to alleviate the symptoms of renal fibrosis (Kolesnyk et al., 2010; Abbas et al., 2011; Zhou et al., 2020). Despite these treatments, the prognosis of renal fibrosis is still poor. Hence, there is an urgent need for the development of clinically effective therapies to treat renal fibrosis and reduce incidence and mortality.

Zhenwu decoction (ZWD) is a classic prescription in traditional Chinese medicine (TCM), composed of five herbs, namely, *Aconitum carmichaeli Debx*., the radix of *Paeonia tacti lora Pall*., *Poria*, *Atractylodes macrocephala Koidz*., and *Zingiber officinale Roscoe*. It possesses a range of bioactivities, such as renoprotective effect, diuresis, anti-inflammation, antioxidant, anti-tumor, and lipid-lowering effects (Cai et al., 2010; Wu et al., 2016; Liu et al., 2017). In the clinic, ZWD has been widely used to treat CKD for more than 1,000 years. Our previous study found that ZWD exhibited an efficient renoprotective effect by reducing oxidative lesions and regulating energy metabolism in unilateral ureteric obstruction (UUO) rats (Li et al., 2018). Clinical renal injury indicators (e.g., Scr, BUN, fibronectin, type III procollagen, and CysC) and pathological changes (HE staining and Masson trichrome staining) showed favorable results with ZWD for renal fibrosis. However, TCM is characterized by its complex composition and complicated mechanism. The absence of an appropriate research method leads to the fact that the mechanisms of most TCMs are difficult to reveal. At present, the specific mechanism of ZWD on the progression of renal fibrosis remains unclear.

The gut flora in the body plays a vital role in the treatment of oral drugs entering the body. The intestinal microbiota transformation of drug active components and the targeted regulation of intestinal microbiota by drugs have become important discoveries in life sciences (Liang et al., 2019). Exploring the role and mechanism of TCM through gut microbiota has become a hotspot in modern medical research. Ever increasing evidence supports that intestinal dysbiosis is associated with various kidney diseases, such as renal fibrosis, CKD, acute kidney injury, diabetic kidney disease, and glomerular nephritis (Vaziri et al., 2013; Lu et al., 2020; Yang et al., 2020; Zhu et al., 2021). Indeed, there may be a bidirectional relationship between intestinal function and renal function. Bacterial translocation leads to damage of intestinal barrier function and systemic inflammation, thereby accelerating the progression of renal fibrosis. Meanwhile, renal fibrosis also affected the structure of intestinal microbiota and contributed to flora dysbiosis. Consequently, a close interconnection has been proposed between the gut and kidney, which is known as the gut–kidney axis (Khoury et al., 2017; Chen et al., 2019). The association between fecal microbiome and serum metabolomics in IgA nephropathy rats and the intervention effect of ZWD have been reported (Li et al., 2021). However, whether ZWD attenuates renal fibrosis through modifying the gut microbiota remains to be elucidated.

In this study, three independent experiments (drug interventions in prophylactic, early, and disease progression periods) were performed on UUO rats to confirm the therapeutic effect of ZWD administration in different stages of renal fibrosis. Meanwhile, accurate detection of microbiota changes caused by drug intervention, but not by renal fibrosis, is of great significance for effectively elucidating the mechanism of action of ZWD. The aim of this study was to investigate the renal protective role of ZWD in UUO rats and its possible mechanisms based on the theory of the gut–kidney axis.

## Materials and methods

### Materials and reagents

*Aconitum carmichaeli Debx*. (Batch No. 190450121; Origin: Sichuan, China), the radix of *Paeonia tacti lora Pall*. (Batch No. 181200279; Origin: Anhui, China), *Poria* (Batch No. 1812002; Origin: Yunnan, China), *Atractylodes macrocephala Koidz*. (Batch No. 1903001; Origin: Zhejiang, China), and *Zingiber officinale Roscoe* (Batch No. 190423; Origin: Guangdong, China) were obtained from the Guangdong Provincial Hospital of Chinese Medicine. Enalapril maleate (EM) was provided by Jiangsu Pharmaceutical Co., Ltd., Cystatin C (CysC), serum creatinine (Scr), and blood urea nitrogen (BUN), serving as indicators of kidney injury, were measured by using biochemical kits purchased from Shanghai Enzyme-linked Biotechnology Co., Ltd. The serum levels of inflammatory factors and immunoglobulins, including interferon-γ (IFN-γ), interleukin-1β (IL-1β), interleukin-4 (IL-4), tumor necrosis factor-α (TNF-α), immunoglobulin A (IgA), immunoglobulin G (IgG), and immunoglobulin M (IgM), were assessed by using commercial ELISA kits following the manufacturers’ instructions (Shanghai Enzyme-linked Biotechnology Co., Ltd., Shanghai; China). The hematoxylin and eosin (HE) staining kit was obtained from Wuhan Boster Biological Technology, Ltd.

### Preparation of Zhenwu decoction

The preparation of ZWD was referred to previous report (Li et al., 2018). The formula consists of *Aconitum carmichaeli Debx*., the radix of *Paeonia tacti lora Pall*., *Poria*, *Atractylodes macrocephala Koidz*., and *Zingiber officinale Roscoe* at a ratio of 3:3:3:2:3. *Aconitum carmichaeli Debx*. was first washed and decocted with seven volumes of pure water (w/v) for 1.5 h. The radix of *Paeonia tacti lora Pall*., *Poria*, *Atractylodes macrocephala Koidz*., and *Zingiber officinale Roscoe* were macerated with seven volumes of pure water (w/v) for 20 min; mixed with the decoction of *Aconitum carmichaeli Debx*.; decocted for 1.5 h; and then filtered. The filtrate was concentrated to a concentration of 3.33 g raw materials per milliliter, placed in a sterile bottle, and stored at −80°C. After the experiment, the voucher specimen (herbal and concentrate) was numbered and deposited at the Guangdong Provincial Hospital of Chinese Medicine (No. 190709).

### Animals and drug administration

Male Sprague–Dawley (SD) rats (*n* = 108) weighing 160 ± 20 g were purchased from the Laboratory Animal Centre at Southern Medical University (Guangzhou, China, animal-qualified certificate: 44002100019613). The experimental protocol was approved by the ethics committee of the Guangdong Provincial Hospital of Chinese Medicine and performed according to the guide for care and use of laboratory animals.

**FIGURE 1:**
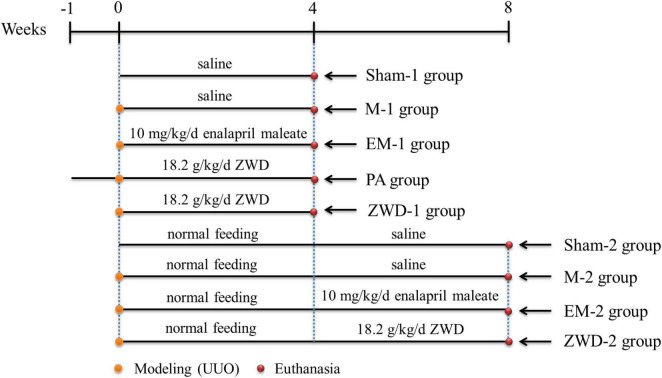
Schematic of the animal experiment and sampling points.

The animals were kept under standard laboratory conditions (temperature 18–23°C; humidity 45–55%; 12-h light/12-h dark cycle) with food and water *ad libitum*. Renal fibrosis was induced in the rats with UUO. According to euthanasia time (4 or 8 weeks after surgery), the rats were randomly divided into two experimental time points, and the detailed grouping criteria are shown in Figure 1. At the first time point, the rats were randomly divided into five groups: sham operation group (sham-1, *n* = 12), model group (M-1, *n* = 12), positive control group (EM-1, *n* = 12; 10 mg/kg/d EM was administered on the 2nd day after modeling), prophylactic treatment group (PA, *n* = 12; 18.2 g/kg/d ZWD was administered for 7 days before modeling), and ZWD treatment group (ZWD-1, *n* = 12; 18.2 g/kg/d ZWD was administered on the 2nd day after modeling). Sham-1 and M-1 groups were administered with normal saline. This step of the experiment lasted 4 weeks after modeling, and the rats were euthanized 24 h after the last administration. At the second time point, the rats were allowed to return to normal feeding without drug administration for 4 weeks to model successfully and then began the administration of the drug for 4 weeks. The rats were randomly divided into four groups: sham operation group 2 (sham-2, *n* = 12), model group 2 (M-2, *n* = 12), positive control group 2 (EM-2, *n* = 12; 10 mg/kg/d, EM was administered on the 28th day after modeling), and ZWD treatment group 2 (ZWD-2, *n* = 12; 18.2 g/kg/d ZWD was administered on the 28th day after modeling). This step of the experiment lasted 8 weeks after modeling, and the rats were euthanized 24 h after the last administration. Finally, the samples of plasma, urine (from metabolic Cages), feces, and kidney were collected for further analyses.

### Hematoxylin and eosin staining

HE staining was performed by following the routine protocol. In brief, part of the left kidney was fixed in 4% paraformaldehyde, dehydrated by gradient ethanol, paraffin-embedded, then sectioned at 4 μm thickness, and stained with HE staining. Images were captured under an optical microscope (Olympus Optical Co., Ltd., Tokyo, Japan). Renal interstitial fibrosis lesions were characterized by the degree of changes to the tubules and glomerulus. The sections were examined for the presence or absence of interstitial fibrosis, interstitial edema, tubular dilation, glomerular atrophy, congestion of periglomerular capillaries, and cell vacuolization. It was scored according to the following criteria: 1 = normal; 2 = mild change (<30%), 3 = moderate change (30–70%), and 4 = severe change (>70%) of the kidney tissue showing abnormalities. The scores for the changes in the tubules and glomerulus were summed to show the overall damage in the kidney.

### 16S rDNA microbial profiling analyses

Total genomic DNA was extracted from fecal samples using a DNA stool kit according to the instructions. The V3–V4 hypervariable region of the 16S rRNA sequence was amplified by PCR with specific primers 338F (5′-ACTCCTACGGAGGCAGCAGCAG-3′) and 806R (5′-GGACTACHVGGTWTCTAAT-3′). After being quantified with QuantiFluor™ fluorometer (Promega, Milano, Italy), purified PCR amplicons were mixed in equal amounts, and connected through sequencing joints. Finally, a sequencing library was constructed, and the sequencing was performed on Illumina HiSeq PE250 (Illumina, CA, United States). The amplicon sequencing data were submitted to the NCBI SRA (SubmissionID, SUB11291689; BioProject ID, PRJNA823644).

### Statistics analysis

Statistical analysis was carried out using SPSS 18.0 software (SPSS, Inc., United States) and GraphPad Prism software 8. First of all, the assumptions of normality and homogeneity of variance were first tested, and the data were presented as mean ± standard deviation for continuous variables with a normal distribution, and as counts and percentages for categorical variables. Second, the independent sample *t*-test or one-way ANOVA was used to analyze the differences among groups for continuous measures. Differences with *P*-values less than 0.05 were considered to have statistical differences, and *P*-values less than 0.01 were considered to have significant differences. All probability values were two-sided.

16S rDNA gene sequence was processed using the Quantitative Insights into Microbial Ecology software (QIIME 2, 2020.02 release)^1^ (Bolyen et al., 2019). Demultiplexed paired-end reads were imported into QIIME 2, and the dada2 plugin (Callahan et al., 2016) was applied to quality trimming, sequence denoising, clustering to amplicon sequence variants, dereplication, and removal of chimera sequences, and default parameters were used to generate filtered feature tables and representative sequences. Alpha-diversity and beta-diversity were investigated running QIIME 2 core diversity script and group significance tests. Community diversity and richness were estimated by α-diversity, utilizing the Goods_coverage, Shannon, Chao1, Ace, Simpson, PD-tree, and Pielou indices. The beta-diversity was computed as weighted UniFrac distances, and differences were visualized with principal coordinates analysis (PCoA) plots. Correlation analysis was performed by Spearman correlation coefficient, and statistical significance was accepted as FDR < 0.05 and *P*-value < 0.05.

## Results

### Zhenwu decoction modulated the renal phenotype and improved kidney function indices

**FIGURE 2:**
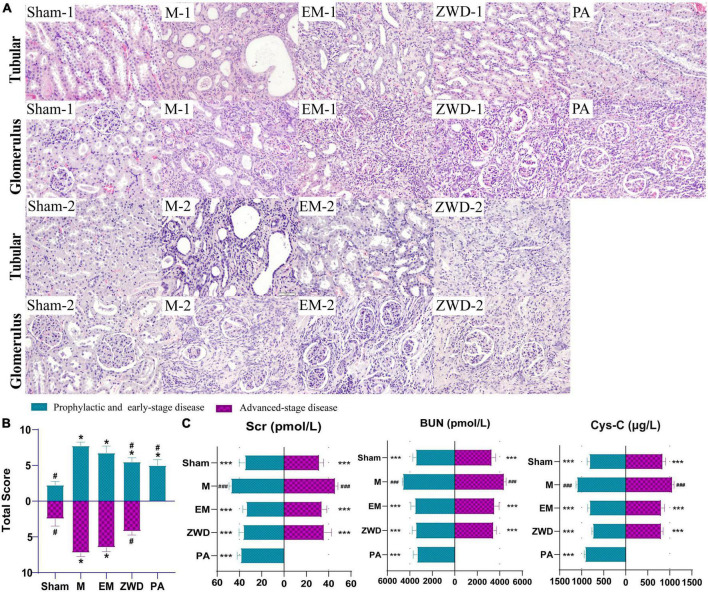
**(A)** Representative HE staining images of the rat left kidney in each group (magnification, × 200). **(B)** Pathological scoring of kidney tissue. **(C)** Comparison of renal parameters (Cys-C, Scr, and BUN) under different interventions. Compared with the sham operation group, *P < 0.05, ***P < 0.001; compared with the model group, ^#^*P* < 0.05, ^###^*P* < 0.001.

The morphological changes in the renal tubules and glomerulus of all experimental groups were observed by HE staining (magnification, 200×). As presented in Figure 2A, the renal tubules were arranged tightly, and the structure of glomerulus was clear and intact in sham-1 and sham-2 groups. In the M-1 and M-2 groups, significant damage in the renal was observed, as evidenced by tubular dilation, structure collapse, and glomerulus and vascular congestion accompanied by infiltration of inflammatory. The overall pathological features in the EM-1 and EM-2 groups were ameliorated as compared with those in the M-1 and M-2 groups. By contrast, in the ZWD treatment group by different administration schedules (PA, ZWD-1, and ZWD-2), tubular dilation and regular glomerulus were markedly improved, and the renal tubular structure was clear and complete compared with the rat model group of the same time. The degree of changes to the structure of the tubule and glomerulus was assessed using the scoring system as previously described (Figure 2B). The model groups (M-1 and M-2) exhibited significantly higher total scores than the sham-1 and sham-2 groups. After ZWD treatment, the total score of the three groups was decreased.

As important indexes of renal fibrosis, concentrations of Cys-C, BUN, and Scr were detected to evaluate renal function. As shown in Figure 2C, unilateral ureter ligation in rats led to renal damage, with a significant increase in serum Cys-C, SCr, and BUN in the model groups. Compared with the model groups, the concentrations of Cys-C, SCr, and BUN in the positive drug and ZWD treatment groups were all significantly decreased (*p* < 0.001). The effects of the three ZWD administration groups were comparable, with no significant difference in levels of Cys-C, SCr, and BUN, demonstrating that three different administration schedules of ZWD had a similar protective effect against renal fibrosis.

### Changes in species diversity were assessed by alpha-diversity and beta-diversity analyses

**FIGURE 3:**
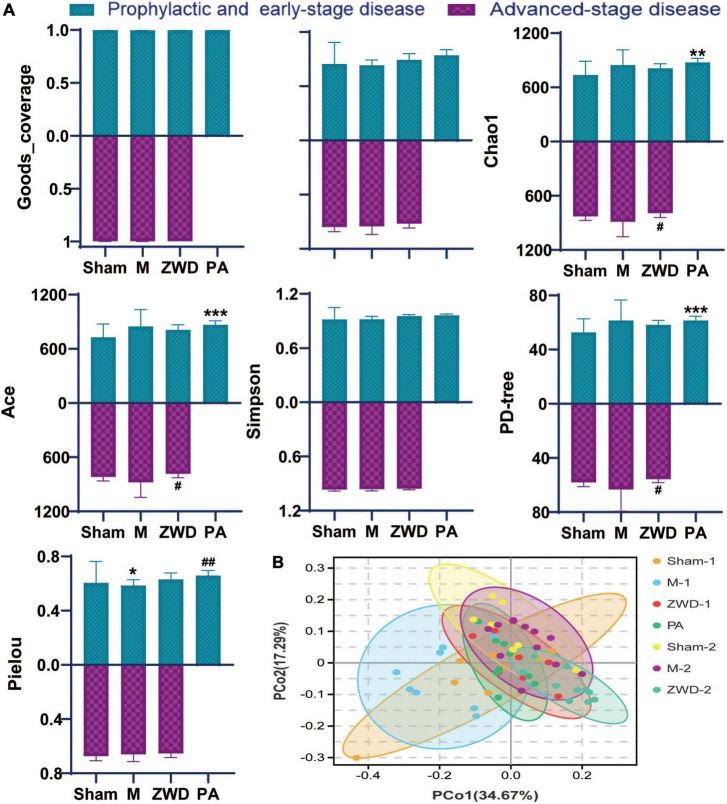
**(A)** Alpha-diversity bar charts between the seven groups, including goods_coverage, Shannon, Chao1, Simpson, Ace, PD-tree, and Pielou indexes. **(B)** Principal coordinates analysis (PCoA). Compared with the sham operation group, *P < 0.05, **P < 0.01, ***P < 0.001; compared with the model group, ^#^*P* < 0.05, ^##^*P* < 0.01.

Goods_coverage was the sequencing depth index, representing the real situation of gut microbiota and reflecting if the sample selection of this experiment was reasonable. Goods_coverage index closer to 1 indicates sufficient sequencing depth (Figure 3A). Microbial species diversity and microbial species evenness were assessed using analytical indices, including Shannon, Chao1, Simpson, Ace, PD-tree, and Pielou. For most diversity indicators (Shannon, Chao1, Simpson, Ace, and PD-tree), no statistically significant differences were observed between the sham operation group and the model groups. Based on the Pielou index, community evenness was lower in the model group. Compared with the M group, the species diversity of the PA group was significantly higher, and both Shannon diversity and Pielou evenness indices showed significant differences between the two groups. No significant difference was found in the Simpson indices among these groups. Overall, these results reveal that rat UUO induced a significant decrease in community evenness compared with that in the sham operation group, and ZWD intervention could generally increase the species diversity of gut microbes. Among them, species richness of rats administrated by ZWD for 7 days before the modeling group was significantly increased compared with modeling rats.

Dissimilarities between samples were conducted by the principal coordinate analysis based on weighted UniFrac distances (Figure 3B). A closer sample distance indicates a more similar species composition structure between the samples. As observed in the PCoA plot, there were obvious alterations in the overall structure and composition of the intestinal microbiota in the model groups compared with the sham operation groups. The differences between PA, ZWD-1, and ZWD-2 groups are not obvious, meaning that all the ZWD-administered groups had similar microbial compositions.

### Zhenwu decoction altered the composition of gut microbial community

**FIGURE 4:**
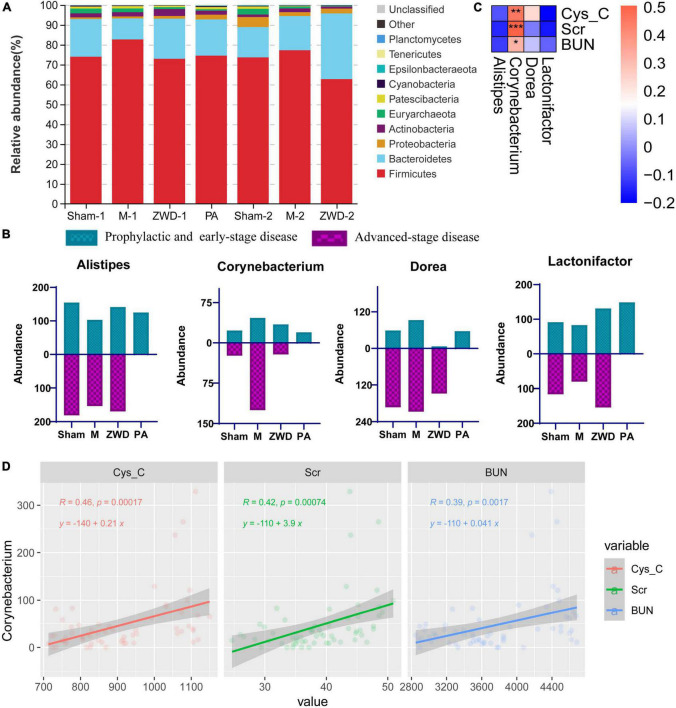
**(A)** Stacked column graph representing the relative abundance of gut bacterial taxa at the phylum level in the different groups. **(B)** Gut microbiota differences at the genus level among each groups. **(C)** Spearman correlation analysis of three renal function indicators (Cys-C, Scr, and BUN) and four differential genera. Color intensity represents the magnitude of correlation; *P < 0.05, **P < 0.01, ***P < 0.001. **(D)** Regression analysis between *Corynebacterium* and three renal function indicators.

Stacked bar charts were used to show the mean relative abundance change in the intestinal microbiota among each group at the species level. At the phylum level, the dominant species were *Firmicutes*, *Bacteroidetes*, *Proteobacteria*, and *Actinobacteria*, which accounted for more than 90% of the intestinal microbiota. The relative abundance of *Firmicutes* increased, while *Bacteroidetes* decreased in response to the model group compared with the sham operation group. ZWD administration significantly reversed the aforementioned changes (Figure 4A). At the genus level, the same variation tendency of *Alistipes*, *Corynebacterium*, *Dorea*, and *Lactonifactor* was observed among three different ZWD administration groups (Figure 4B). This suggested that ZWD treatment was associated with changes in the abundance of these key microorganisms at the genus level, which warranted further study.

### Correlations between intestinal microflora and kidney fibrosis indices

To investigate the relationship between intestinal microflora and kidney fibrosis, correlation analysis was performed between key genera and kidney function indices (Cys-C, Scr, and BUN). As shown in Figure 4C, *Corynebacterium* abundance, which was decreased in the ZWD groups, presented a significant positive correlation (Spearman, FDR, *p* < 0.05) with Cys-C, Scr, and BUN. These results revealed that a decrease in *Corynebacterium* was related to kidney function improvement and had potential value for renal fibrosis treatment. Meanwhile, regression analysis was used to further investigate the relationship. The results demonstrated a strong linear relationship between *Corynebacterium* and kidney function indices (Figure 4D), which further validated the aforementioned results.

### Zhenwu decoction exhibited beneficial anti-inflammatory and immunomodulatory effects on unilateral ureteric obstruction rats

**FIGURE 5:**
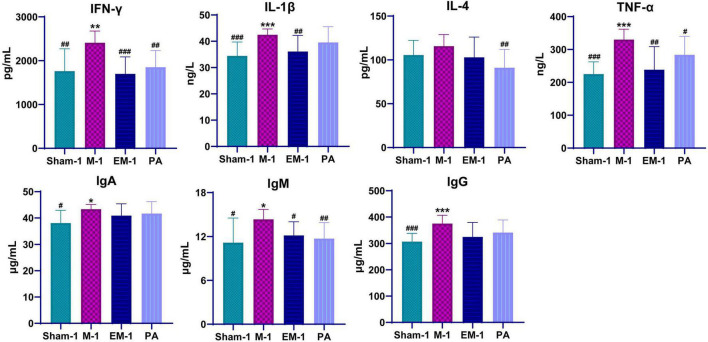
Dynamic changes of serum inflammatory cytokines and immunoglobulins before and after ZWD treatment. Compared with the sham operation group, *P < 0.05, **P < 0.01, ***P < 0.001; compared with the model group, ^#^*P* < 0.05, ^##^*P* < 0.01, ^###^*P* < 0.001.

To evaluate inflammatory response, inflammatory cytokines (INF-γ, IL-1β, IL-4, and TNF-α) and immunoglobulin (IgA, IgM, and IgG) were measured by ELISA. Compared with rats in the sham operation groups, those who received modeling had significantly higher serum levels of INF-γ, IL-1β, IL-4, TNF-α, IgA, IgM, and lgG (Figure 5). This phenomenon had a significant callback trend after positive drug and ZWD administration. These results further confirmed the beneficial anti-inflammatory and immunomodulatory effects of ZWD following kidney fibrosis.

## Discussion

The progression of renal fibrosis is a multi-step and multi-factorial dynamic pathological process involving interstitial macrophage infiltration, interstitial fibroblast-to-myofibroblast transdifferentiation, and ECM accumulation (Humphreys, 2018). At present, the treatment of renal fibrosis is still a clinical challenge. ZWD, as a classical prescription for warming up the yang and promoting diuresis, has been widely used to treat CKD for more than 1,000 years. In this study, we used ZWD as research subjects, explored the protective effects of ZWD against renal fibrosis in different intervention periods, and elucidated complex crosstalk between the gut and the kidney. The results indicated the treatment effect was comparable between the three ZWD protocols, and significant improvement was observed in the renal phenotype and kidney function indices. The preventive/protective mechanism of ZWD may be explained based on the anti-inflammatory potential of important pharmacodynamic ingredients. For example, total glucosides of peony (paeoniflorin, albiflorin, and oxypaeoniflorin) derived from the root of *Paeonia lactiflora* are believed to be the main bioactive compounds for anti-inflammation *in vitro* and *in vivo*. Wu et al. (2009) showed that total glucosides of peony treatment ameliorated early renal injury *via* the inhibition of expression of ICAM-1, IL-1, TNF-α, and 3-NT in the kidneys of diabetic rats. Another important class of compounds is flavonoid, which has a good antioxidant activity, such as quercetin. A study conducted by Lu et al. (2018) suggested that quercetin was shown to be able to inhibit M1 macrophage polarization *via* NF-κB and IRF5 signaling, thereby contributing to the inactivation of upstream signaling TLR4/Myd88. Moreover, quercetin also inhibited M2 macrophage polarization and reduced excessive accumulation of the extracellular matrix and interstitial fibrosis *via* the TGF-β/Smad signaling pathway. The protective effect of active compounds of ZWD on kidney injury showed that the administration of ZWD could reduce UUO-induced inflammation and fibrosis.

Renal fibrosis can cause intestinal microbial dysbiosis and is accompanied by chronic inflammation and aberrant immune activation (Yang et al., 2020). In this study, the richness, diversity, and evenness of the bacterial communities were investigated according to the abundance of OTUs based on 16S DNA sequencing approach. The results suggested that rat UUO induced a significant decrease in community evenness compared with that in the sham operation group, while ZWD administration exhibited a callback effect on species richness. ZWD could modulate intestinal bacterial composition, and it significantly reduced the relative abundance of *Firmicutes* and increased the abundance of *Bacteroidetes* when comparing the model group, resulting in a lower *Firmicutes/Bacteroidetes* ratio. Previous research reported that changes in *Firmicutes* and *Bacteroidetes* contribute to hosting metabolic disorders through changing the metabolism of energy, lipid and amino acids, and inflammatory response (Ley et al., 2006; Turnbaugh et al., 2006; Koliada et al., 2017). The *Firmicutes/Bacteroidetes* ratio was tightly associated with microbiota metabolic capacity and inflammation, which is often used as an indicator of the interstitial microbiota balance.

**FIGURE 6:**
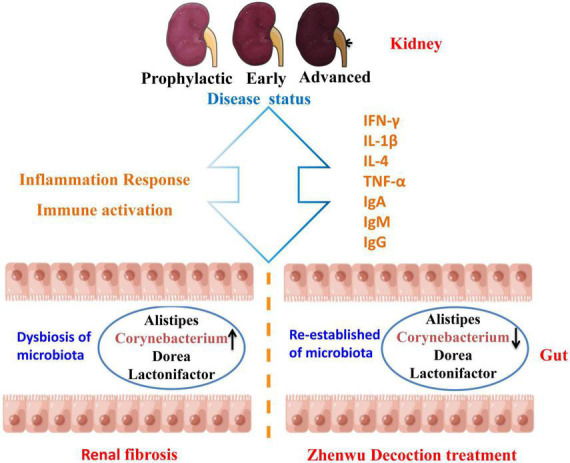
Diagram of the mechanism of action of ZWD against renal fibrosis. ↑ represents upregulation, and ↓ represents downregulation.

At the genus level, the same consistent variation trend of *Alistipes*, *Corynebacterium*, *Dorea*, and *Lactonifactor* was observed in three different ZWD administration groups (Figure 6), which suggested that ZWD treatment was associated with alterations in the abundance of these key microbial species at the genus level and warranted further investigation. In addition, correlation analysis was conducted between the differential flora and renal fibrosis indicators. It is worth noting that *Corynebacterium* presented a significant positive correlation (Spearman, FDR, *p* < 0.05) with the concentration of Cys-C, Scr, and BUN in plasma. Regression analysis further validated the aforementioned results.

In the genus *Corynebacterium*, many species are involved in human and animal diseases, causing a variety of infections, such as bacteremia, arthritis, osteomyelitis, meningitis, endocarditis, breast abscess, peritonitis, wound infections, and prosthetic joint infections (Belmares et al., 2007; Zasada and Mosiej, 2018; Dragomirescu et al., 2020). Garcia et al. (2020) reported a series of cases in which *Corynebacterium striatum* presented an opportunistic infection in patients with end-stage kidney disease. *Corynebacterium striatum* was originally considered a contaminant in many of these cases and was only subsequently considered to be the likely culprit. *Corynebacterium urealyticum* is the most common opportunistic pathogen, which is a cause of urinary tract infection and encrusting cystitis or pyelitis (López-Medrano et al., 2008; Soriano and Tauch, 2008; Sakhi et al., 2020). Local inflammation caused by encrustations and pyelonephritis may lead to deterioration of renal function. It also causes other infections but mainly in patients with urological diseases. If left unchecked or unrecognized, the disease process can progress to renal compromise, resulting in end-stage renal failure. Chronic allograft dysfunction is a potential complication in kidney transplant recipients. Wu et al. (2018) indicated that *Corynebacterium* was the only genus found more in patients with chronic allograft dysfunction than in controls. These pieces of clinical evidence suggested that *Corynebacterium* could lead to a dysregulation of inflammatory feedback mechanisms and promoted the development of CKD and progression to end-stage kidney disease. It has been well recognized that changes in the intestinal microbiota play an important role in the activation of the host immune system and may cause systemic inflammation response, while inflammation is a critical feature for the initiation and progression of renal fibrosis.

Persistent inflammation promotes the fibrosis process, resulting in chronic pathology and eventually end-stage kidney disease. The inflammatory response of the kidney is characterized by glomerular and tubulointerstitial infiltration of inflammatory cells, like macrophages, dendritic cells, and T cells (Meng et al., 2014). However, the progression of inflammation to fibrosis is driven by transition of the macrophage infiltrate from classically activated M1 phenotype to alternatively activated M2 phenotype. The pro-inflammatory M1 macrophages generate a large number of pro-inflammatory cytokines (TNF-α, IL-1β, IL-1, and IL-6), nitric oxide, and reactive oxygen species in response to kidney injury. By contrast, the profibrotic M2 macrophages secrete anti-inflammatory cytokines like IL-4/IL-13, TGF-β, IL-10, and glucocorticoid activation (Meng, 2019). Macrophages contribute to renal fibrosis *via* several mechanisms: M2 macrophages produce profibrotic factors (e.g., TGF-β1, FGF-2, and PDGF), which promote myofibroblast proliferation and extracellular matrix overproduction. In a profibrotic microenvironment, macrophages stimulate fibronectin and collagen production in fibroblasts or transdifferentiate into collagen-producing fibrocytes. When those tissue repair mediators secreted in excess and maintained at high levels, they are also key inducers of kidney fibrosis (Sepe et al., 2022). Profibrotic mediators activate a profibrotic signaling network through interconnection and cross-talking among multiple signaling pathways, such as TGF-β, Wnt, NF-κB, RAS, integrin/ILK, and mTOR (Wang et al., 2021). Compared with rats in the sham operation groups, those who received modeling had significantly higher serum levels of INF-γ, IL-1β, IL-4, TNF-α, IgA, IgM, and lgG (Figure 5). This phenomenon had a significant callback trend after positive drug and ZWD administration. The results further proved the beneficial anti-inflammatory and immunomodulatory effects of ZWD following kidney fibrosis.

*Alistipes* is a member of *Bacteroidetes*. Studies have shown that *Alistipes* has a correlation with fibrosis, cancer, cardiovascular disease, mood disorders, and other potential diseases (Campion et al., 2019; Parker et al., 2020). Earlier studies have illustrated that *Alistipes* can produce anti-inflammatory metabolites, which would promote the differentiation of anti-inflammatory Treg/Tr1 cells in the gut of mice, and *Alistipes* can also protect mice suffering from the effects of dextran sulfate sodium (Dziarski et al., 2016; Li et al., 2016). Liu et al. (2022) confirmed that the increased relative abundance of fecal *Alistipes* has a causal relationship to decreased triglyceride concentration by Mendelian randomization analyses. *Dorea* and *Lactonifactor* are within the phylum *Firmicutes*. There is a positive correlation between *Lactonifactor* and urinary nitrogen excretion (Alves et al., 2021). The difference in urinary N excretion mainly related to urine volume and urinary N concentration. ZWD warmed up the kidney and improved the Qi transformation effect, which induce diuresis in order to alleviate edema, thus leading to the increase in urine flow and urinary N excretion. However, our experiments did not validate whether ZWD had an effect against renal fibrosis by directly impacting or remodeling the gut microbiota. This is the limitation of this study and needs to be examined in future studies.

## Conclusion

The re-establishing of the intestinal microbiota imbalance may provide a novel prevention or treatment strategy for renal fibrosis. This study confirmed the therapeutic effect of ZWD on renal fibrosis in different disease stages and clarified the complex crosstalk between the gut and the kidney. ZWD ameliorated inflammation and immune response in the kidneys by regulating the composition of intestinal flora, including *Corynebacterium*, *Alistipes*, *Dorea*, and *Lactonifactor*. The most striking of them was *Corynebacterium*, which may be a key flora of ZWD against renal fibrosis.

## Data availability statement

The datasets presented in this study can be found in online repositories. The names of the repository/repositories and accession number(s) can be found below: NCBI BioProject—PRJNA823644.

## Ethics statement

This animal study was reviewed and approved by the Ethics Committee of Guangdong Provincial Hospital of Chinese Medicine.

## Author contributions

LD: animal experiment, data analysis and interpretation, and manuscript writing. YZ: animal experiment and data acquisition. SJ and LW: data acquisition and analysis. XZ: sample collection and manuscript revision. SY: study conception and design and manuscript revision. XX: study conception and design. SL: study conception and design and manuscript writing. All authors contributed to the article and approved the submitted version.

## Footnotes

1
http://qiime.org/
